# Assessment of a novel 32-channel phased array for cardiovascular hybrid PET/MRI imaging: MRI performance

**DOI:** 10.1186/s41824-019-0061-7

**Published:** 2019-08-22

**Authors:** Adam Farag, R. Terry Thompson, Jonathan D. Thiessen, John Butler, Frank S. Prato, Jean Théberge

**Affiliations:** 1 Lawson Health Research Institute, Imaging Division, 268 Grosvenor St., Rm E5-118, PO Box 5777, STN B, London, ON N6A 4V2 Canada; 2 Department of Medical Biophysics, Western University, London, ON Canada; 3 Department of Medical Imaging, Western University, London, ON Canada; 4 St. Joseph’s Health Care, Diagnostic Imaging, London, ON Canada

**Keywords:** PET/MRI, Cardiac imaging, Phased array, Parallel imaging

## Abstract

**Background:**

Cardiovascular imaging using hybrid positron emission tomography (PET) and magnetic resonance imaging (MRI) requires a radio frequency phased array resonator capable of high acceleration factors in order to achieve the shortest breath-holds while maintaining optimal MRI signal-to-noise ratio (SNR) and minimum PET photon attenuation. To our knowledge, the only two arrays used today for hybrid PET/MRI cardiovascular imaging are either incapable of achieving high acceleration or affect the PET photon count greatly.

**Purpose:**

This study is focused on the evaluation of the MRI performance of a novel third-party prototype 32-channel phased array designed for simultaneous PET/MRI cardiovascular imaging. The study compares the quality parameters of MRI parallel imaging, such as g-factor, noise correlation coefficients, and SNR, to the conventional arrays (mMR 12-channel and MRI-only 32-channel) currently used with hybrid PET/MRI systems. The quality parameters of parallel imaging were estimated for multiple acceleration factors on a phantom and three healthy volunteers. Using a Germanium-68 (Ge-68) phantom, preliminary measurements of PET photon attenuation caused by the novel array were briefly compared to the photon counts produced from no-array measurements.

**Results:**

The global mean of the g-factor and SNR_g_ produced by the novel 32-channel PET/MRI array were better than those produced by the MRI-only 32-channel array by 5% or more. The novel array has resulted in MRI SNR improvements of > 30% at all acceleration factors, in comparison to the mMR12-channel array. Preliminary evaluation of PET transparency showed less than 5% photon attenuation caused by both anterior and posterior parts of the novel array.

**Conclusions:**

The MRI performance of the novel PET/MRI 32-channel array qualifies it to be a viable alternative to the conventional arrays for cardiovascular hybrid PET/MRI. A detailed evaluation of the novel array’s PET performance remains to be conducted, but cursory assessment promises significantly reduced attenuation.

## Introduction

Hybrid imaging systems combining positron emission tomography (PET) and magnetic resonance imaging (MRI), namely PET/MRI scanners, are unique in providing both functional and intrinsically registered anatomical information from both PET and MRI simultaneously. PET/MRI systems are therefore highly advantageous for multimodality studies, which can improve the characterization and grading of metabolically active tumors using^18^F-fluoro-deoxy-glucose (FDG) (Catana et al. 2012; Camici et al. 2008), while superimposing such activity on anatomical images with the superior soft-tissue contrast of MRI to identify active inflammation (White et al. 2013). In addition, by combining perfusion tracers such as ^13^N-labeled-Ammonia (^13^NH_3_) with FDG, the PET/MRI has also been shown to be successful in cardiovascular imaging where left/right ventricular function and myocardial perfusion and blood flow in surrounding vessels of the heart can be quantified (Camici et al. 2008; Nensa et al. 2018; Nensa et al. 2014).

Since the emergence of whole-body PET/MRI systems, several technical challenges have been identified and commonly reported around the accurate quantification of the 511 keV annihilation photons detected in PET images (Pichler et al. 2008a; Pichler et al. 2008b; Rausch et al. 2017; Wagenknecht et al. 2013). The greatest challenge, apart from respiratory and cardiac motion, is the accurate correction of PET counts due to attenuation within materials located between the radioactive source and the PET detectors. Attenuation of 511 keV photons is caused by the presence of the patient body (tissues, air cavities, blood, and bones) and the scanner hardware in the PET field-of-view (FOV). Hardware, such as the patient bed, audio communication system, and radio frequency (RF) resonators, have the most attenuating effect and cause scattering or simply block the gamma rays from reaching the PET detectors (Kartmann et al. 2013; MacDonald et al. 2011; Tellmann et al. 2011). However, RF phased arrays are crucial for MRI parallel imaging as they achieve the shortest scan time with the highest spatial and temporal resolutions, and their use, particularly for cardiovascular MRI, is an essential part of the standard of care. The advantage of parallel imaging with high acceleration factor up to 4 in one-dimension for cardiac MRI was proven to be beneficial for imaging (Wintersperger et al. 2006). Therefore, a phased array with 32-channels is highly desired. Although a few dedicated PET/MRI RF phased arrays have been developed for brain (Anazodo et al. 2016 ; Sander et al. 2014) and breast (Dregely et al. 2014) imaging, no phased array, to our knowledge, has been developed for PET/MRI cardiovascular imaging. Currently, MRI-only research systems offer phased arrays with up to 128-channels for cardiovascular imaging (Schmitt et al. 2008), while clinical PET/MRI systems offer up to 12-channels. Although commercially available, PET/MRI arrays are typically restricted to a lower number of channels to reduce attenuation of gamma rays. Nevertheless, PET/MRI arrays have been reported to cause variation of the standardized uptake value (SUV) ranging from 18 to 60% closer to the array, if the attenuation correction (AC) is not included during the PET image reconstruction (Fürst et al. 2012; Ouyang et al. 2014; Paulus et al. 2012).

Meanwhile, developing a dedicated PET/MRI phased array with a higher number of channels is technically challenging, leading researchers to focus on the approach of correcting the attenuation of the currently available MRI arrays (Eldib et al. 2015; Ferguson et al. 2014; Frohwein et al. 2018; Kartmann et al. 2013). The common approach to correct for attenuation of an RF phased array is to generate a hardware AC map, also known as a μ-map, produced from a CT scan at a specific tube voltage (Carney et al. 2006; Patrick et al. 2017). The process of generating a hardware-AC map is normally conducted by the scanner manufacturers for all vendor-provided hardware, prior to delivery of the scanner. AC maps are accurately included during PET image reconstruction for fixed rigid hardware, such as the patient table and rigid RF arrays. For more accurate AC of flexible RF arrays, fiducial markers can be added to guide the registration of the hardware AC map with the PET image (Kartmann et al. 2013).

Although, RF arrays with a lower number of channels are adequate for most oncologic applications, simultaneous PET/MRI for cardiovascular imaging can still benefit from faster parallel imaging using a dedicated PET/MRI RF 32-channel phased array prospectively designed for minimal PET attenuation. It is, therefore, important to investigate alternative approaches to minimize the attenuation caused by a high-density phased array for cardiovascular PET/MRI imaging. In this work, we evaluate a third-party dedicated 32-channel phased array optimized for parallel imaging of the heart with PET/MRI. The quality parameters for parallel imaging, such as the geometry factor (g-factor), SNR, and noise correlation coefficients, are compared to the two commercially available and currently used, MRI-only 32-channel and the PET/MRI 12-channel arrays.

The reduction in PET signal due to attenuation caused by the candidate array was briefly examined and reported. Although this work focusses on the MRI performance of the array, the equally important PET performance of the candidate array will be compared in detail to the existing arrays and will be reported in a separate manuscript. Only a cursory evaluation of PET performance will be reported herein. We hypothesize that if the MRI quality parameters of the PET/MRI 32-channel array are similar with those produced by MRI-only 32-channel array, and if global gamma ray attenuation of the PET/MRI 32-channel array is less than that of the mMR 12-channel array, the MRI performance of the PET/MRI 32-channel array would be acceptable for hybrid/simultaneous PET/MRI cardiovascular imaging.

## Materials and methods

### Description of the arrays

The PET/MRI phased array consists of two parts; a posterior and an anterior, allowing the patient to be scanned in head-first, supine position. The RF elements were arranged to cover the entire heart region and can be connected to the scanner at four ports. The ports are arranged, with two ports for the anterior array, and two ports for the posterior array, allowing each part of the array to be used independently. The anterior and posterior part of the 32-channel arrays has 16 elements each, arranged in a 4 × 4 fashion to maximize acceleration factor in directions within the coronal plane. The flexible body mMR 6-channel array (Siemens Healthcare Limited, Erlangen, Germany) elements are arranged in 3 × 2 fashion, which is similar in arrangement to the spine matrix array (Siemens Healthcare Limited, Erlangen, Germany). Therefore, the flexible body mMR 6-channel array was combined with six elements from the posterior spine matrix mMR array formulating a set of mMR 12-channel array used in this work.

In this study, a commercially available cardiac MRI-only 32-channel array (In-Vivo Corporation, Gainesville, FL, USA) was used, while the prototype novel PET/MRI 32-channel (developed in 2016) was provided for assessment by a local company (Ceresensa. Inc., Canada).

### MRI phantom imaging

For this study, a balanced steady-state free precession (balanced-SSFP or TrueFISP in vendor’s nomenclature) pulse technique was selected due to its ability to acquire sub-second scan time per slice and its sensitivity to high fluid-tissue contrast (Haacke et al. 1990). All MRI acquisitions were performed on a 3.0T PET/MRI system (Biograph mMR Software Version VE11P, Siemens Healthineers, Erlangen, Germany).

**Fig. 1:**
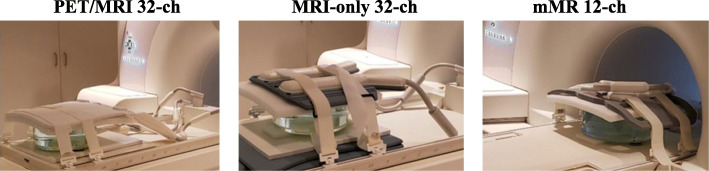
Phantom experiment setup for the three arrays PET/MRI 32-channel, MRI-only 32-channel, and mMR 12-channel. Position of isocenter were kept unchanged from one acquisition to the other

All three arrays operated with posterior and anterior parts, making the condition of the measurements identical as seen in Fig. 1. Phantom imaging data were acquired using a standard cylindrical acrylic container (OD = 28 cm) filled with a solution of both NiSO_4_·6(H_2_O) and NaCl in distilled water. To mimic the location of the heart in a patient, the distance between the anterior and posterior array elements were kept to approximately 27 cm and the 12 cm high cylindrical phantom was centered between them, leaving the elements approximately 7.5 cm away from the surface of the phantom. To examine the parallel imaging capabilities of each array, a single 2D-slice at the center of the phantom in the coronal plane was acquired using the manufacturer’s 2D TrueFISP sequence. Multiple acquisitions were performed with phase encoding in left-right (LR) direction, for different acceleration factors ranging from *R* = 1 to *R* = 6. A second set of acquisitions similar to the above was performed, changing the phase encoding into the foot-head (FH) direction, for the same number of acceleration factors. The image reconstruction utilized the generalized autocalibration partially parallel acquisitions (GRAPPA) technique (Griswold et al. 2002), with 64 reference lines for all accelerations. The 2D TrueFISP-GRAPPA parameters for the LR and FH encoding were BW = 440 Hz/pixel, FOV = 253 × 253 mm, spatial resolution of 1.3 × 1.3 × 8.0 mm^3^, flip angle = 50°, and TE/TR = 2.40/4.79 ms. For each acceleration factor *R*, noise data was also acquired with the same parameters except the RF amplitude was set to zero. The above two sets of coronal acquisitions were repeated two times on the phantom for each array, with a total of 64 acquisitions for each array.

A reflected power test was performed to ensure that there is no undue change to the RF field transmitted by the integrated body resonator due to poor decoupling from the receiver array (Schmitt et al. 2008). A low percent difference in reference voltage between the “with-array” and “no-array” conditions indicates that no undue amount of transmitted energy is absorbed within the receiver array. The reference voltage was determined by the scanner’s automated RF calibration procedure and represents the voltage necessary to obtain a 180° flip angle using a 1 ms square RF pulse. This test was performed only on the PET/MRI 32-channel array, since it is not licensed or approved by a third-body regulator.

### In-vivo imaging

For in-vivo imaging, acquisitions were performed on three healthy volunteers, recruited with written informed consent according to a research ethics protocol approved by the Research Ethics Board (protocol ID 6319). In addition to the same pulse sequences that were performed in the phantom acquisitions, ECG-triggered 2D TrueFISP cine images were acquired on a single breath-hold with axial double-oblique orientation and a four-chamber view of the heart. The imaging parameters of the cine MRI with activated GRAPPA for *R* = 2 (anterior-posterior phase encoding) were TE/TR = 1.58/36.3 ms, 25 segments, spatial resolution of 1.0 × 1.4 × 6.0 mm^3^, FOV = 253 × 300 mm, flip angle = 50°, and BW = 930 ± 16 Hz/pixel. For the three volunteers, the average beat-to-beat interval was 906 ± 50 ms. Each volunteer was fitted with MRI-compatible ECG electrodes, so that the data is retrospectively ECG-gated. Volunteers were imaged in the head-first supine position, with a total acquisition time of under 10 s in 13 heart beats. Each volunteer was imaged consecutively with all three arrays under the same conditions and using the same imaging parameters as described above. The full duration of an imaging session for one volunteer with the three arrays was under 60 min.

### Data processing and analysis

For phased arrays, SNR is more likely to be overestimated if measured from the magnitude of the image, due to bias of the Rician-distributed noise in the image magnitude (Henkelman 1985; Pruessmann et al. 1999). The noise measured by a phased array is influenced by the overlapping of each element and cannot be treated as a single source of noise, i.e., a fraction of the noise observed by each element is correlated noise and must be accounted for via noise correlation coefficient to avoid overestimation. Notably, the mMR 12-channel array utilizes OEM-specified combiner chip, while both MRI-only and PET/MRI arrays do not. Therefore, using raw data (k-space) for signal and noise was necessary to perform appropriate and fair comparison and identical reconstruction. Two techniques were introduced (Pruessmann et al. 1999; Robson et al. 2008), by which SNR of phased arrays can be estimated in a pixel-by-pixel fashion. Both techniques have addressed the geometric overlapping of array elements (known as g-factor). In this work, the g-factor was computed from both the estimated noise covariance, which was computed from noise correlation coefficients of the array elements in Appendix A (Pruessmann et al. 1999), and sensitivity map (spatial element sensitivity) which was estimated off-line. The formulas used to compute sensitivity, g-factor, and SNR parameters are described by Eqs. (1), (2), and (3) in Pruessmann et al. (1999) and presented here for convenience.

1 $$ {S}_{pj}={S}_j\ast {r}_{\mathrm{p}} $$

2 $$ {\mathrm{g}}_p=\sqrt{{\left({\left({S}^H{\Psi}^{-1}\mathrm{S}\right)}^{-1}\right)}_{p,p}\ {\left({S}^H{\Psi}^{-1}S\right)}_{p,p}} $$

Where *S* is the complex sensitivity matrix, Ψ the noise correlation coefficient, *r* is the aliased *p* pixel position, and *j* is the array element number. The g-factor-based SNR_g_ was estimated on a pixel-by-pixel fashion for multiple acceleration factors *R* = 2 to *R* = 6 from the fully sampled image SNR_0_, which was found by the difference method (Price et al. 1990).

3 $$ {\mathrm{SNR}}_{\mathrm{g}}=\frac{{\mathrm{SNR}}_0}{{\mathrm{g}}_p\sqrt{R}\ } $$

For the two phantom experiments, global mean, from masked images, and standard deviation (SD) of the SNR_0_, SNR_g_, and g-factor were calculated at each *R* value independently for both one-dimension (phase encoding LR or FH) and two-dimensions (phase encoding is in both LR and FH). The relationship between mean SNR_g_ and inverse g-factor as functions of *R* were examined and compared for the three arrays. Mean and SD of the noise correlation coefficients were estimated excluding the self-correlated coefficients (diagonal values of the matrix). The percentage difference of the quality parameters for the MRI-only and PET/MRI 32-channel arrays were estimated using Eq. (4), where *v*_1_ is the PET/MRI array parameter value and *v*_2_ is the MRI-only array parameter value. For the interpretation of the percentage difference, a criterion was established in which the absolute function in the formula was ignored allowing directional estimates. Instead, the measured parameters for the MRI-only array were subtracted from those of the PET/MRI array. Hence, a negative percentage difference would indicate a better performance of the PET/MRI array for the g-factor parameter. All quality parameters of the parallel imaging and data analysis for each array were computed using Matlab 9.3.0 (The MathWorks, Natick, MA, USA).

4 $$ \%\mathrm{difference}=\frac{v_1-{v}_2}{\ 0.5\ \left({v}_1+{v}_2\right)}\ 100\% $$

### PET activity test

To estimate PET photon attenuation, two-point Dixon acquisitions were performed prior to the PET acquisition, with and without the array, on the mMR Ge-68 daily quality control (QC) phantom (Siemens, Healthineers, Erlangen, Germany). The Dixon acquisition consisted of a 3D dual-echo spoiled gradient sequence with the following parameters: TE/TR = 1.23/3.96 ms, slice thickness = 3.1 mm, flip angle = 9°, and FOV = 312 × 500 mm. The phantom was mounted following the procedure used in the daily QC, where neither the patient table nor array were present in the bore of the PET/MRI system during the Dixon acquisition. An 8-min PET acquisition was carried out immediately after the Dixon while the array was placed around the phantom. Placing the array took no more than 2 min; therefore, only the last 6 min of the data were used for PET image reconstruction. The resultant data was labeled as “no-array, no-table” and was used as a base line to compare to PET/MRI array. The same acquisitions were repeated on the phantom but with the array’s anterior part placed on the top of the QC phantom, while the array’s posterior part was placed below the QC phantom. Mean of PET counts per seconds (CPS) from the acquisitions, without applying AC maps, were estimated from the central transaxial slice of the phantom for each PET acquisition.

## Results

The voltage recorded for 180° RF pulse with the volunteer in the scanner, but without the PET/MRI 32-channel array, was 562 V. It was 598 V with the volunteer loading the array in the scanner, resulting in only 6% difference. This percentage difference of the reference voltages is indicative of the decoupling inefficiency of the array and shows that the coupling between the array and the body resonator is minimal. In this case, the PET/MRI 32-channel array shows 94% decoupling efficiency.

### MRI phantom imaging

**Fig. 2:**
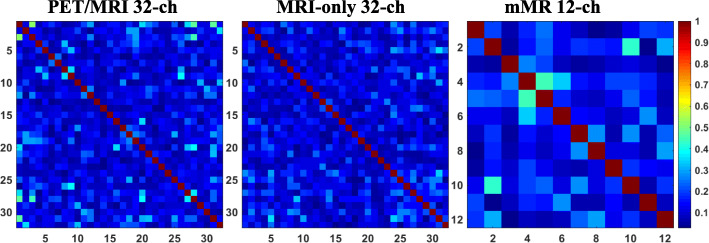
Noise correlation coefficients matrix for each of the three arrays; PET/MRI 32-channel, MRI-only 32-channel, and mMR 12-channel. The scale of the color bar represents the correlation coefficients values, which could also be reported as correlation percentage. The average noise correlation coefficients (excluding the matrix diagonal) for the PET/MRI array was measured to be < 0.13 (< 13% correlation)

Table 1 summarizes the MRI quality parameter means and SDs, such as SNR_0_, SNR_g_ sensitivity-based g-factor, and inverse g-factor as estimated for all arrays at all acceleration factors, in 1D (LR or FH) and 2D. The table also includes the mean and SD of the noise correlation coefficients for all arrays. Noise correlation coefficient matrices representing between-channel coupling for the three arrays are graphically represented in Fig. 2. Each array produced a mean noise correlation coefficient of less than 0.2 (20% correlation). For example, noise correlation means at *R* = 2, excluding self-correlated elements (diagonal), were 10%, 11%, and 15% for the MRI-only 32-channel, PET/MRI 32-channel, and mMR 12-channel arrays, respectively.

**Table 1:** Mean ± SD of parallel imaging quality parameters (noise correlation coefficients, SNR_0_, SNR_g_, and 1/g) computed for the three arrays. The table lists the parameters for different phase encoding direction in 1D (LR or FH) and in 2D (LR and FH). Note the similar or better performance of the PET-MRI 32ch array compared to the MRI-only 32-channel array

	PET/MRI 32-channel					MRI-only 32-channel					mMR 12-channel				
	*R =* 1	*R* = 2	*R* = 3	*R* = 4	*R* = 6	*R* = 1	*R* = 2	*R* = 3	*R* = 4	*R* = 6	*R* = 1	*R* = 2	*R* = 3	*R* = 4	*R* = 6
Noise correlation coefficient	0.110 ± 0.004	0.110 ± 0.002	0.120 ± 0.011	0.120 ± 0.004	0.120 ± 0.003	0.090 ± 0.003	0.100 ± 0.004	0.100 ± 0.001	0.110 ± 0.001	0.110 ± 0.002	0.150 ± 0.001	0.150 ± 0.021	0.150 ± 0.011	0.150 ± 0.001	0.160 ±0.001
Encoding															
1D-LR															
SNR_0_	965 ± 112	–	–	–	–	871 ± 98	–	–	–	–	749 ± 90	–	–	–	–
SNR_g_	–	608 ± 57	428 ± 38	271 ± 25	88 ± 22	–	581 ± 71	386 ± 66	229 ± 34	55 ± 18	–	488 ± 58	247 ± 26	99 ± 7	24 ± 3
g-factor	1	1.06 ± 0.05	1.25 ± 0.16	1.81 ± 0.54	7.03 ± 4.59	1	1.06 ± 0.05	1.32 ± 0.18	2.13 ± 0.79	15.41 ± 13.56	1	1.05 ± 0.04	2.10 ± 1.07	6.94 ± 5.42	41.75 ± 39.56
1/g-factor	1	0.98 ± 0.04	0.82 ± 0.10	0.60 ± 0.16	0.23 ± 0.15	1	0.95 ± 0.04	0.77 ± 0.10	0.53 ± 0.17	0.16 ± 0.15	1	0.96 ± 0.04	0.59 ± 0.25	0.28 ± 0.24	0.08 ± 0.13
1D-FH															
SNR_0_	929 ± 107	–	–	–	–	816 ± 96	–	–	–	–	655 ± 83	–	–	–	–
SNR_g_	–	648 ± 53	467 ± 35	279 ± 26	73 ± 19	–	549 ± 41	365 ± 59	216 ± 18	52 ± 12	–	443 ± 56	224 ± 23	90 ± 6	21 ± 2
g-factor	1	1.01 ± 0.02	1.16 ± 0.11	1.82 ± 0.59	8.97 ± 6.27	1	1.06 ± 0.05	1.32 ± 0.17	2.12 ± 0.79	15.44 ± 13.54	1	1.05 ± 0.04	2.10 ± 1.06	6.93 ± 5.40	43.77 ± 41.75
1/g-factor	1	0.99 ± 0.02	0.87 ± 0.08	0.60 ± 0.17	0.19 ± 0.16	1	0.95 ± 0.04	0.77 ± 0.10	0.53 ± 0.17	0.16 ± 0.15	1	0.96 ± 0.04	0.59 ± 0.25	0.27 ± 0.24	0.08 ± 0.13
2D	*R* = 2 × 2	*R* = 2 × 3	*R* = 2 × 4	*R* = 3 × 3	*R* = 3 × 4	*R* = 2 × 2	*R* = 2 × 3	*R* = 2 × 4	*R* = 3 × 3	*R* = 3 × 4	*R* = 2 × 2	*R* = 2 × 3	*R* = 2 × 4	*R* = 3 × 3	*R* = 3 × 4
SNR_g_	572 ± 23	404 ± 21	237 ± 16	324 ± 19	192 ± 14	623 ± 26	406 ± 19	233 ± 15	330 ± 16	198 ± 13	427 ± 21	196 ± 15	64 ± 14	132 ± 16	42 ± 9
g-factor	1.05 ± 0.03	1.23 ± 0.14	1.97 ± 0.66	1.59 ± 0.36	2.52 ± 0.94	1.06 ± 0.03	1.35 ± 0.18	2.28 ± 0.88	1.72 ± 0.42	2.73 ± 1.14	1.12 ± 0.06	2.75 ± 1.89	10.16 ± 5.84	5.41 ± 4.86	18.51 ± 13.03
1/g-factor	0.95 ± 0.03	0.82 ± 0.09	0.56 ± 0.16	0.66 ± 0.13	0.45 ± 0.15	0.94 ± 0.03	0.75 ± 0.10	0.50 ± 0.16	0.61 ± 0.13	0.42 ± 0.14	0.89 ± 0.05	0.50 ± 0.24	0.19 ± 0.22	0.34 ± 0.21	0.13 ± 0.16

**Fig. 3:**
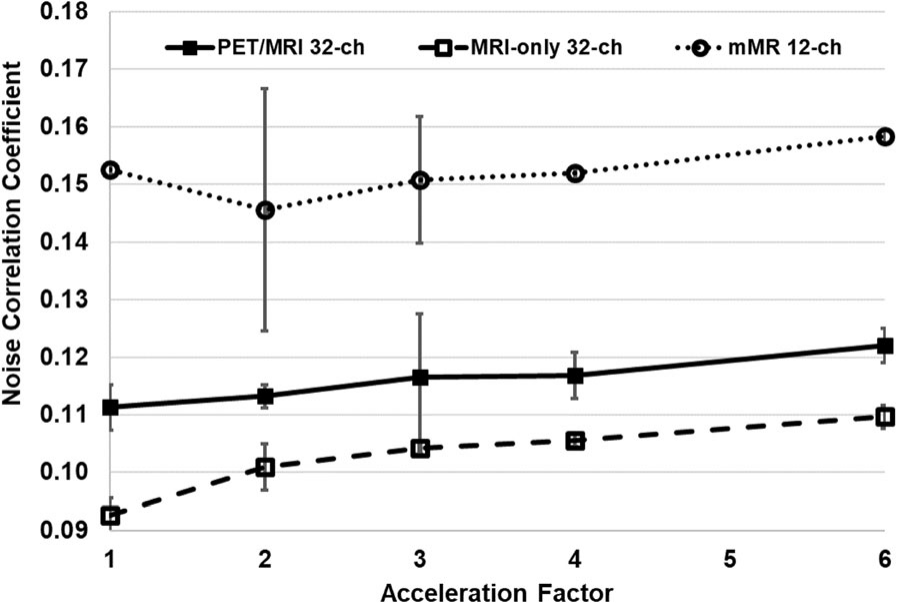
Mean and SD of the noise correlation coefficients shown as a function of acceleration factor for the three arrays. Of note: the mMR 12-channel array mean noise correlation shows a drop at *R* = 2, with SD of 0.021 (2.1%). The MRI-only 32-channel shows the lowest values, yet noise correlation coefficients increases by 0.019 (1.9%) from *R* = 1 to *R* = 6, while the PET/MRI 32-channel array has shown an increase by 0.012 (1.2%)

Figure 3 displays means and SDs of the noise correlation coefficients for the three arrays as a function of the acceleration factor, with the mMR 12-channel array producing the largest standard deviation (2.1%) of all three arrays.

**Fig. 4:**
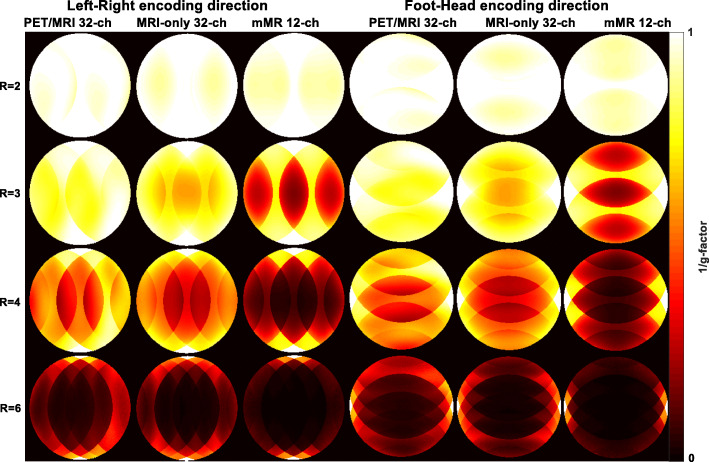
1/g factor maps for the three arrays with *R* = 2 to *R* = 6 in both LR and FH phase encoding direction. Notice a substantial noise amplification beyond *R* = 2 for the mMR 12-channel array in comparison to the two 32-channel arrays

Figure 4 presents measured inverse g-factors maps for 1D acceleration (phase encoding in LR and FH direction) for all arrays. The 1/g-factor maps of the PET/MRI 32-channel array are compared to the two commercial arrays for each acceleration factor and produced the lowest mean g-factor and, hence, the lowest noise amplification of the three arrays, as recorded in Table 1. For example, at acceleration factor *R* = 3 in the LR direction, the mMR array produced a mean g-factor of 2.10 compared to 1.32 for the MRI-only 32-channel array and 1.25 for the PET/MRI 32-channel array. A similar pattern of g-factor was also observed when estimated with encoding in the FH direction.

Using percentage difference formula number [4] with parameters in Table 1, one can compare any parameters of one array to another. Beyond acceleration factor of *R* = 2 in either encoding directions, the PET/MRI 32-channel array shows more than 30% improvement in g-factor and SNR_g_ compared to the mMR array. The PET/MRI 32-channel array mean g-factor for *R* = 3 in the FH encoding direction was improved relative to the LR phase encoding direction by 7%, while the MRI-only 32-channel array showed no changes in mean g-factors at *R* = 3 according to phase encoding direction.

**Fig. 5:**
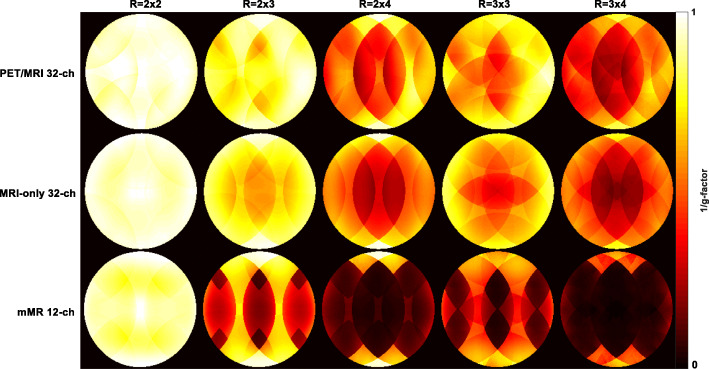
1/g factor maps in the case of 2D acceleration with *RR* = 2 × 2 to *RR* = 3 × 4. The PET/MRI 32-channel array achieves the least noise amplification with max of 0.97 at *RR* = 3 × 3 in comparison to 0.88 for the MRI-only array, while the mMR 12-channel array produced the maximum noise amplification of 0.83

**Fig. 6:**
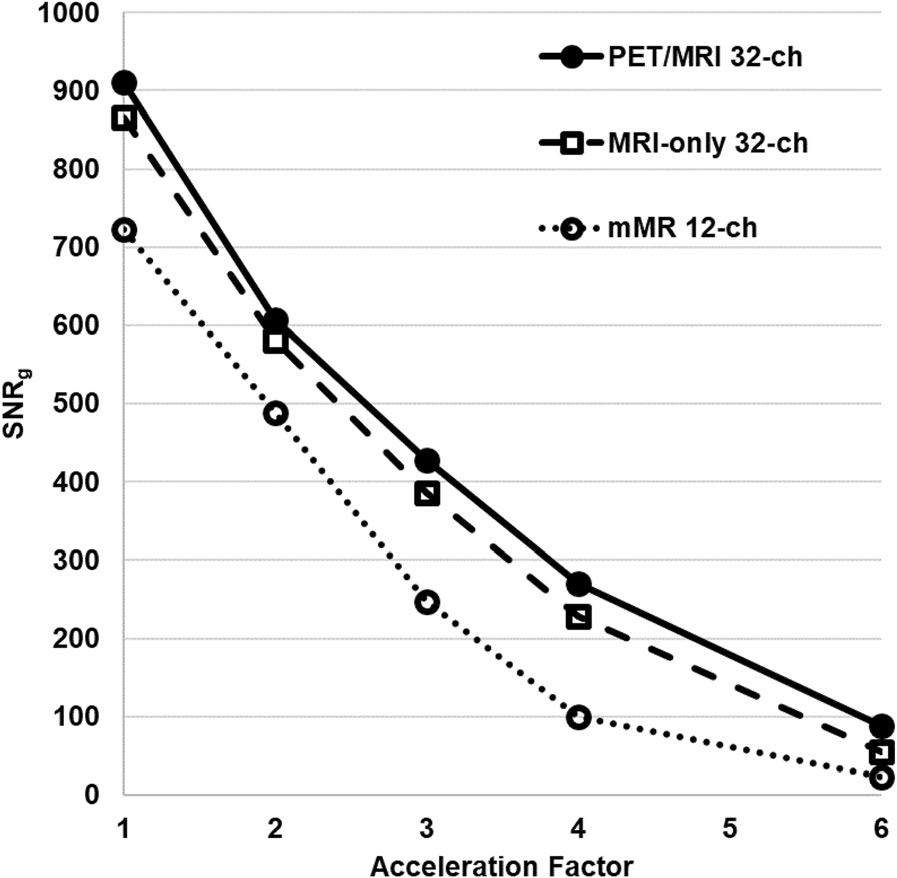
Estimated SNR_g_ as a function of acceleration factors from *R* = 1 to *R* = 6, with GRAPPA reconstruction. The estimated SNR_g_ shown here follows the theory and is in agreement with expected profile as first proposed by (Griswold et al. 2002)

Figure 5 presents the 1/g-factor maps for selected 2D acceleration factors *R*_LR_ × *R*_FH_ = 2 × 2, 2 × 3, 2 × 4, 3 × 3, and 3 × 4. In Fig. 5 and Table 1, the 2D acceleration is shown to be possible with the PET/MRI 32-channel for up to 3 × 3 with only a SNR loss of less than half of that of the mMR 12-channel array, and 8% better than the MRI-only 32-channel array. Table 2 focuses on the percentage differences of the parallel imaging quality parameters between the two 32-channel arrays for the case of L-R encoding direction. The percentage difference between the non-accelerated (*R* = 1) SNR_0_ for both arrays was 10.3%, while the SNR_0_ measured from the PET/MRI array at *R* = 2 was better than the MRI-only array by approximately 19%. The percentage differences in the case of mean g-factor for *R* = 1 and *R* = 2 were found to be negligible, while for accelerations factors *R* > 2, the mean g-factor of the PET/MRI array was more than 5% over that measured from the MRI-only array, as seen in Table 2. The mean and SD of SNR_g_, as a function of acceleration factor for the three arrays, are displayed in Fig. 6.

**Table 2:** The percent difference of parallel imaging quality parameters (left-to-right encoding direction) comparing both 32-channel arrays

PET/MRI 32-ch and MR-only 32-ch percentage difference (%)					
	*R* = 1	*R* = 2	*R* = 3	*R* = 4	*R* = 6
SNR_0_	10.3	19.2	11.7	− 1.1	−0.2
SNR_g_	5.2	4.4	10.4	16.9	45.6
Noise correlation coefficients	18.4	11.6	11.2	10.2	10.7
Max g-factor	0	6.0	− 5.1	− 16.4^a^	− 107.0^a^
Mean g-factor	0	0.5	− 5.5	− 15.9^a^	− 74.7^a^
Mean 1/g-factor	0	− 0.3	5.9	11.9	39.3

^a^Negative values in this table indicate advantage of the PET/MRI 32ch array over the MRI-only 32ch array

### MRI in vivo imaging

**Fig. 7:**
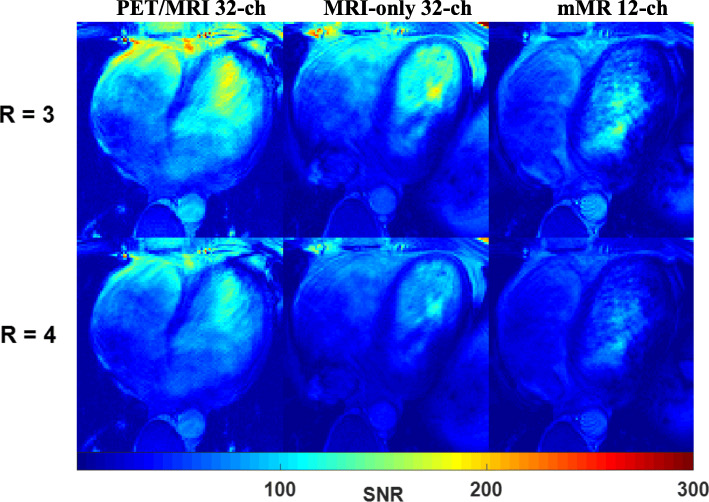
In vivo maps of SNR_g_ (i.e., including noise amplification considerations) comparison of the three arrays, using a single center-slice 2D TrueFISP image acquired at *R* = 3 and *R* = 4, with left-right encoding. The SNR maps show greater degradation of the SNR for the mMR 12-channel compared to the 32ch arrays at higher acceleration factors

**Fig. 8:**
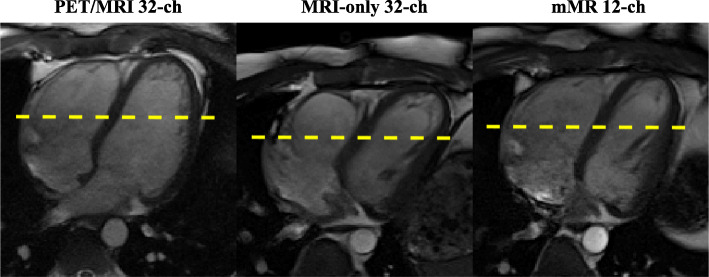
The first of 25 four-chamber view images acquired with a 2D TrueFISP cine sequence on a male volunteer with acceleration factor of 2 (*R* = 2)

**Fig. 9:**
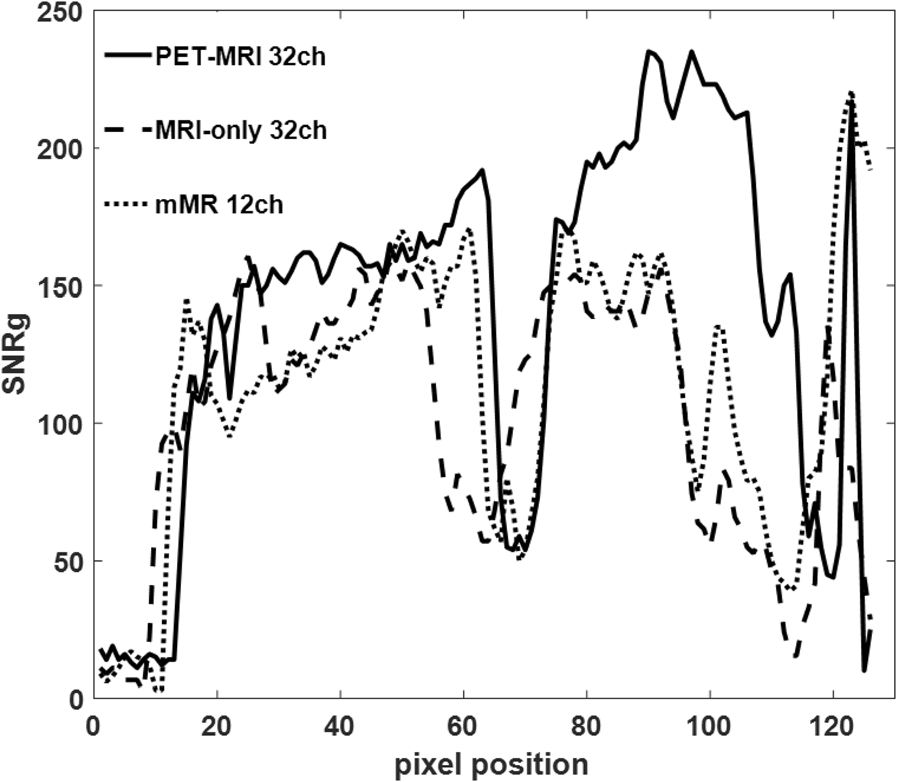
A centre line (dashed line in Fig. 8) profile of the heart showing SNRg generated by the three arrays using a 2D TrueFISP cine four-chamber view. The scanning plane/orientation is not identical, and the line had to be centered in the heart as much as possible for each acquisition above. The SNR_g_ profile of the PET/MRI array (solid line) is higher than the other arrays

The SNR_g_ pixel-by-pixel maps from in vivo acquisitions (single centre-slice 2D TrueFISP, cine disabled), at 1D acceleration factor *R* = 3 and *R* = 4, were derived by utilizing elements sensitivity and are presented in Fig. 7. At the mid-ventricular level and up to the apex, the effect of the noise amplifications at *R* = 3 (and higher) are comparable for the 32-channel arrays and they outperform the mMR 12-channel array. A higher SNR_g_ from the in vivo acquisitions (2D TrueFISP cine 4-chamber view) on a male volunteer was achieved using the PET/MRI 32-channel array (Fig. 8). The line profile of the SNR_g_ across the center of a four-chamber view of the heart was approximately 15% higher for the PET/MRI 32-channel array over the two other conventional arrays (Fig. 9).

### PET imaging

**Fig. 10:**
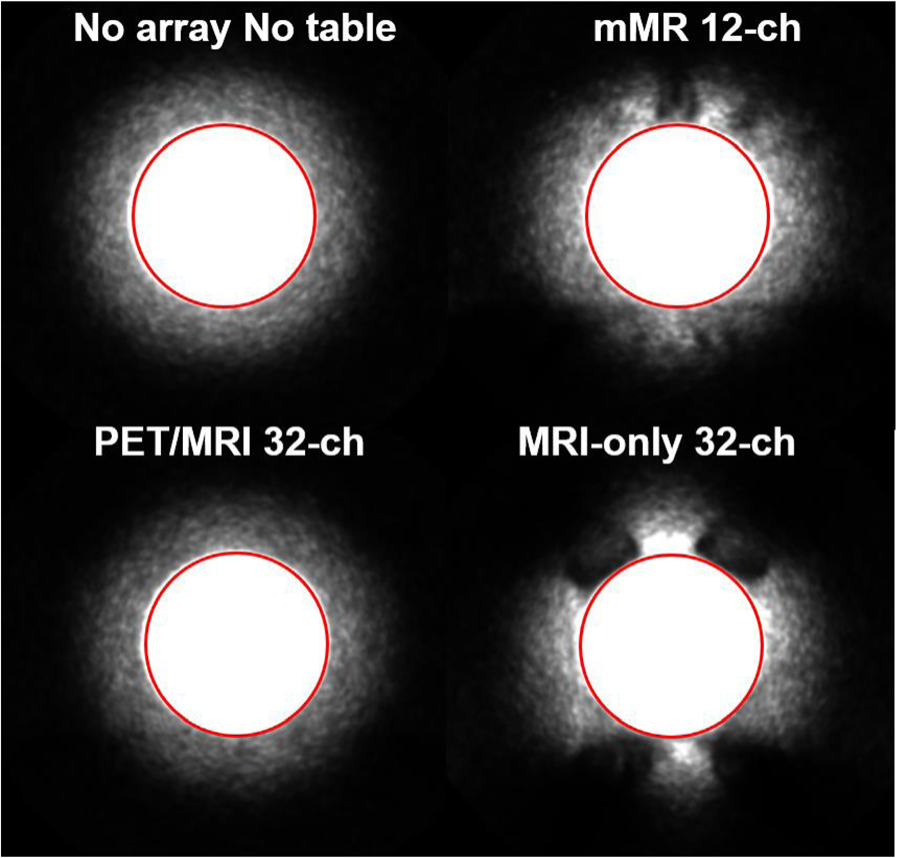
PET counts per second (CPS) map of the middle slice of the Ge-68 phantom for each array separately and reference (“no-array, no-table”). The global mean count per seconds was measured from the region contoured by the red circle. The figure is windowed to accentuate the hardware attenuation artifacts visible immediately outside the phantom. All images used identical windowing

Figure 10 compares center-slice of the PET activities acquired on the Ge-68 phantom for the three arrays and the “no-array, no-table” measurement. The estimated global mean of the PET counts per second (CPS), from the circled region contouring the phantom, were found to be 557CPS, 534CPS, 436CPS, and 395CPS, for “no-table no-array,” PET/MRI 32-channel array, MRI-only 32-channel array, and mMR 12-channel array respectively. The difference between the PET CPS from “no-table, no-array” to PET/MRI 32-channel array was 4.1%.

## Discussion and conclusion

Overall, the MRI performance assessed by the percentage differences of noise correlation, g-factor, and SNR between the two 32-channel arrays is found to be similar and favored the PET/MRI array greatly at acceleration of *R* = 4 and *R* = 6. The design of this dedicated PET/MRI array has provided superior (> 30%) in all the results over the mMR 12-channel array for acceleration factors greater than 2, theoretically allowing for use of shorter breath-holds, which is often critical in cardiac imaging.

The method used in this work to assess the MRI quality parameters for parallel imaging are commonly used by researchers as reported in Meise et al. (2010), Reeder et al. (2005), Schmitt et al. (2008), Wiggins et al. (2006), and Wintersperger et al. (2006). This work has combined the use of both parallel imaging techniques with high-density arrays to shorten the breath-hold during acquisition which is necessary for cardiovascular imaging. At *R* = 4 using TrueFISP imaging, the shortest breath-hold that could be achieved was approximately 9 s producing 25 images of a single slice.

Unlike the two currently used arrays, the anterior portion of the PET/MRI array is very light and thin and as such does not conform to the subject’s chest under its own weight. This was resolved by using straps to achieve adequate conformity and proximity to the subject’s body. With this design, its elements have the highest proximity to the heart of the three arrays, which benefits penetration depth and results into a better SNR far from the array as demonstrated by Fig. 9.

We recorded lower noise correlation SDs (0.5%) for both 32-channel arrays compared to the mMR 12-channel array, as seen in Fig. 3, which indicates greater stability of the arrays’ noise correlation coefficients as a function of acceleration factor during parallel imaging. One element of the PET/MRI 32-channel array produced a noise coefficient of 61%, and this may have caused the high mean of the coefficients. This could be due to off-resonance tuning of the element which introduced excess noise to the neighboring ones.

We have demonstrated with the phantom results in Figs. 4 and 5 that the 32-channel PET/MRI array produces higher SNR than the other two arrays for 1D and 2D acceleration, which confirms that the array is a strong candidate for use as the MRI receiver array in hybrid PET/MRI cardiovascular imaging. As seen in Table 1, at almost all *R* values, the mean g-factor of the PET/MRI 32-channel array showed better results than those of the other two arrays. The SNR behavior from phantom and in vivo measurements matches those reported in Griswold et al. (2002) regarding parallel imaging theories compared to the effect of acceleration on SNR measured with phased arrays. It was noticed that the estimated SNR_g_ of the MRI-only 32-channel array at *R* = 4 is 17% less than the PET/MRI array, yet it produced the smallest noise correlation coefficient. The reason for this behavior could be due to smaller element size and geometry differences from one array to the other, or alternative reasons may be a suboptimal output gain adjustment or imperfect signal pre-amplification. The parallel imaging quality parameters of the prospectively designed PET/MRI 32-channel array are comparable to the MRI-only array confirming its ability to be employed for 1D acceleration up to *R* = 6, and 2D parallel imaging up to acceleration of 3 × 3.

In conclusion, the PET/MRI 32-channel array prospectively designed for simultaneous PET and MRI demonstrated competitive MRI performance compared to both the 32-channel MRI-only array and the 12-channel PET/MRI array. PET photon attenuation caused by the PET/MRI 32-channel array was measured to be < 5% compared to the no-array PET photon activities. The PET performance will be studied in detail and will be presented in a separate manuscript.

We therefore conclude that the PET/MRI 32-channel array studied here is a viable alternative for simultaneous cardiovascular PET/MRI using parallel imaging. The PET/MRI array can surpass currently used arrays, particularly for high parallel imaging acceleration applications.

## Data Availability

The data that support the findings of this study are available from Lawson Health Research, but restrictions apply to the availability of these data, which were used under license for the current study, and so are not publicly available. Data are however available from the authors upon reasonable request and with permission of Lawson Health Research.

## Abbreviations

AC: Attenuation correction
FDG: ^18^F-fluoro-deoxy-glucose
FH: Foot-head
FOV: Field-of-view
GRAPPA: Generalized autocalibration partially parallel acquisitions
LAC: Linear attenuation coefficients
LR: Left-right
MRI: Magnetic resonance imaging
PET: Positron emission tomography
QC: Quality control
RF: Radio frequency
SD: Standard deviation
SNR: Signal-to-noise ratio
SUV: Standardized uptake value
TrueFISP: Steady-state free precession
UTE: Ultra-short echo time
